# The Influence of Logging-Related Soil Disturbance on Pioneer Tree Regeneration in Mixed Temperate Forests

**DOI:** 10.3390/plants13152149

**Published:** 2024-08-03

**Authors:** Farzam Tavankar, Rachele Venanzi, Mehrdad Nikooy, Angela Lo Monaco, Rodolfo Picchio, Ramin Naghdi

**Affiliations:** 1 Department of Forestry, Khalkhal Branch, Islamic Azad University, Khalkhal 56817-31367, Iran; fa.tavankar@iau.ac.ir; 2 Department of Agricultural and Forest Sciences, Tuscia University, 01100 Viterbo, Italy; venanzi@unitus.it (R.V.); r.picchio@unitus.it (R.P.); 3 Department of Forestry, Faculty of Natural Resources, University of Guilan, Someh Sara P.O. Box 1144, Iran; nikooy@guilan.ac.ir (M.N.); rnaghdi@guilan.ac.ir (R.N.)

**Keywords:** natural regeneration, seedling architecture, seedling biomass, soil compaction, soil penetration resistance

## Abstract

The recovery of soil properties and the proper growth of natural tree regeneration are key elements for maintaining forest productivity after selective logging operations. This study was conducted on the soil properties and natural growth of two pioneer seedling species of alder and maple which were on skid trails in the mixed beech forests of northern Iran. To examine the long-term effects, we randomly selected six skid trails, with two replicates established for each of three time periods since last use (10, 20, and 30 years ago). Random plots 4 m × 10 m in size, three plots on each skid trail and six plots on areas without soil compaction (control), were selected. Measurements included the physical and chemical properties of the soil and the growth, and the architectural and qualitative characteristics of the seedlings. The results showed that all the soil properties of the 10- and 20-year-old skid trails were significantly different from the control area (except for the soil moisture in the 20-year-old skid trail). The 30-year-old skid trail showed values of other soil properties which were not significantly different from the control area, except for the amounts of organic matter and soil nitrogen, which was less than the control. The skid trails had a negative effect on all of the growth, qualitative, and architectural indices of seedlings. The characteristics of seedlings were related to soil characteristics and had the highest correlation with the soil penetration resistance (R-value from −0.41 to −0.63 for stem growth, *p* < 0.05; −0.57 to −0.90 for root growth, *p* < 0.01; and −0.76 to −0.86 for biomass, *p* < 0.01). The correlation coefficient between soil penetration resistance and the Dickson quality index of alder and maple seedlings was, respectively, −0.74 and −0.72, *p* < 0.01. The negative effect of soil compaction on root growth (−27.69% for alder seedlings and −28.08% for maple seedlings) was greater than on stem growth (−24.11% for alder seedlings and −16.27% for maple seedlings). The amount of growth, qualitative, and architectural indices of alder seedlings were higher than that of maple seedlings. Although alder is a better choice as compared to maple seedling in the initial year, the results of our study show that it is recommended to plant both alder and maple on skid trails after logging operations.

## 1. Introduction

Extensive soil damage is a potentially serious threat to the soil ecosystem [1,2]. In recent decades, concerns have been raised about soil protection during logging operations in forests [3,4,5]. Logging operations have been found to cause a considerable degree of forest soil disturbance [6,7,8,9,10]. The use of heavy machinery for timber harvesting operations in forests has increased in the past decades due to their high productivity [11,12,13]. These machines, which weigh 10 to 20 tons during timber extraction, may seriously affect the soil ecosystem, as they cause ruts in the upper soil layers and soil compaction [14,15,16]. These machines have an extended reach, and thus the impact of the effects lasts for a long time [17,18]. That is why forestry activities performed with this type of machinery is considered to be the most harmful [1,16,17,18,19].

A common detrimental effect of timber harvesting is soil compaction caused by heavy machinery [3,4]. Soil compaction reduces coarse pores and total porosity, infiltration capacity, and permeability to water [20,21]. It also increases infiltration resistance [22,23,24]. Compaction creates negative changes in the soil structure and increases the bulk density and strength of the soil such as penetration and shearing resistance. Soil compaction is one of the main concerns of timber harvesting operations in productive forests [3,18,25,26], and especially in the forests of northern Iran [27,28,29]. The timber harvesting operations in these forests is carried out for the most part by the ground logging system. Chainsaw and skidder are the most common type machines used in timber harvesting operations in the ground logging system. The regeneration of these forests occurs naturally and its structure is an unevenly aged high forest. Sustainable production of timber in natural high forests requires a continuous establishment of natural regeneration of trees and sufficient growth of seedlings. The soil bulk density is usually higher in landing sites and skid trails than undisturbed forest soils [30,31]. Soil compaction disrupts soil structure, reducing porosity, aeration, and infiltration [14], which hinders water and air movement, promotes runoff, and increases erosion [9]. Soil compaction during the mechanized timber harvesting in the forest affects the growth and performance of the forest by affecting seed germination and establishment, seedling survival, and growth [1]. Increased resistance to soil infiltration can reduce seedling growth performance in general [32,33,34,35].

Tree seedlings are particularly sensitive to increased resistance to soil infiltration [27]. Increasing resistance to soil penetration reduces the length and penetration of the roots, and thus reduces the absorption of water and nutrients. Seedlings with a less developed root system are more sensitive to soil compaction than mature trees [31]. The survival and growth of different species of tree seedlings ensures the successful regeneration of forest stands, which is the most important step towards achieving long-term sustainability of mixed high forests [35]. In the forests of northern Iran, alder (*Alnus subcordata* C.A. Mey.) and maple (*Acer cappadocicum* Gled.) seedlings are among the first tree species that naturally settle and grow on logging roads after timber harvesting operations [36]. The seedlings of these species are resistant to intense light and are a pioneer species [37]. Alder and maple trees are economically valuable species known for their large stature which can reach a diameter over 90 cm and a height of 30 m in old age [20]. The effects of logging-related soil disturbances on the regeneration of these mixed temperate forests remain largely unexplored.

Sustainable forest management requires specific information on the details of how trees regenerate. This issue is not only important from an ecological perspective, but also has other environmental and social consequences. These are due to the fundamental role of the forest cover to prevent soil erosion and landslides [38,39]. One of the main threats to the sustainability of timber production in naturally managed forests in northern Iran is the insufficient regeneration of timber tree species on skid trails [28,29,30,31,32,33,34]. Disturbance and compaction of the forest soil following logging operations and the traffic of logging machines can lead to a decrease in the long-term productivity of the habitat and potential problems in forest sustainability [3,40].

In the attempt to gather some data for better understanding, the seedlings of alder and maple trees were selected as experimental samples. It is important to know that there is little information about the tolerance and natural recovery of seedlings of different forest tree species to soil disturbance.

Our hypothesis was that plants contribute to the rehabilitation of soil that has undergone compaction during silvicultural operations. Alder and maple are early successional species that colonize compacted soils earlier than other species in mixed broadleaf forests.

The capacity of alder and maple root systems to support seedling growth in compacted soils was uncertain, particularly on skid trails abandoned for 10, 20, or 30 years.

Alder and maple seedlings grown on skid trails were studied to investigate the following:

1. The changes over time in the soil physical and chemical characteristics that influence the growth of seedlings.
2. The ability of alder and maple seedlings to grow on compact forest soils and penetrate them with their roots.
3. The potential use of alder and maple planting to enhance the structural regeneration of compacted forest soils.

As part of this study, a forest management system closely connected to the Continuous Cover Forestry (CCF) was applied to forest operations. Disturbances observed were assessed by the correlation between edaphic parameters and tree renewal. The determination of the recovery time becomes more interesting given the consideration of the level of sensitivity to climate change in these forest ecosystems.

The objectives of this study were (a) to determine the physical and chemical characteristics of the soil of skid trails after 10, 20, and 30 years of logging operations, and (b) to determine the effects of soil compaction on the characteristics of growth, yield, architecture, and quality of alder and maple seedlings.

## 2. Results

The ANOVA results showed that the treatments had a significant effect on all the physical and chemical characteristics of the soil (*p* < 0.01; Table 1). The highest value of soil bulk density and soil penetration resistance occurred in the 10-year-old skid trail, while the lowest value was obtained in the control area. The highest amount of porosity, moisture content, organic matter, nitrogen, and soil acidity was obtained in the control area and the lowest in the 10-year-old skid trail. With the increase in the age of the skid trail from 10 to 20 and 20 to 30 years, the value of soil bulk density decreased by, respectively, −4.76% and −8.33%, and soil penetration resistance decreased by −17.39% and −23.68%, respectively, and the amount of porosity (+4.39% and +6.95%, respectively), moisture (+6.08% and +9.72%, respectively), organic matter (+12.12% and +25.00%, respectively), nitrogen (+18.18% and +50.00%, respectively), and soil acidity (+1.09% and +4.14%, respectively) increased.

Bulk density decreased by about 15% in the 30-year-old skid trails when last used as compared to the 10-year-old ones. However, despite the obvious recovery and the non-significant difference compared to the control, the bulk density after 30 years was still almost 8% higher than in the control. The penetration resistance decreased by approximately 21% in skid trails abandoned 20 years ago compared to skid trails that were abandoned 10 years ago. Considering 30- and 20-year-old disused skid trails, the decrease in penetration resistance was almost 30%.

Except for organic matter, nitrogen, and their ratio, the other soil characteristics in the 30-year-old skid trail were not significantly different from the control area. Except for the value of penetration resistance and organic matter, the other soil characteristics in the 10-year-old skid trail were not significantly different from the 20-year-old skid trail (Table 1).

The ANOVA results showed that all of the physical characteristics of the seedlings of both alder and maple species were affected by the studied treatments considering the last use of the skid trail (Table 2). The best physical characteristics of the seedlings of both alder and maple species were found in the control area, while the lowest were found in the 10-year-old skid trails. The values of all physical characteristics of both alder and maple species in the 10- and 20-year-old skid trails were significantly lower than their values in the control area. The values of stem height, stem length and lateral root length of both alder and maple species in the 30-year-old skid trails were not significantly different from their values in the control area, but the values of length and diameter of the main root and the root penetration depth of both the alder and maple species in the 30-year-old skid trails were significantly lower than their values in the control area. The stem diameter of alder seedlings in the 30-year-old skid trails was not significantly different from the value in the control area, while the stem diameter of maple seedlings in the 30-year-old skid trails was significantly lower than its value in the control area. In the 10-year-old skid trail, among growth characteristics, only root length (main and lateral) showed significant differences between alder and maple seedlings. Furthermore, both the main and lateral root lengths of the alder seedlings were significantly longer than those of maple seedlings. In the 20- and 30-year-old skid trails, with regard to root growth characteristics (length and diameter of the main root and length of the lateral roots), the values of the alder seedlings were significantly higher than those of the maple seedlings. Regarding the root penetration depth, maple and alder showed no significant differences, only in the 20-year-old skid trails. In the control area, the values of all growth characteristics of alder seedlings were significantly higher than the maple seedlings (Table 2).

The results of analysis of variance showed that the above- and belowground organ biomass of alder and maple species was affected by the studied treatments (Table 3). The biomass of the aboveground and belowground organs and the total biomass of both alder and maple species were the highest in the control area, and the lowest were in the 10-year-old skid trails. The amount of stem biomass and root biomass of both species of alder and maple in the 10-year-old and 20-year-old skid trails were not significantly different, but the total biomass of both alder and maple species in the 20-year-old skid trail was higher than its value in the 10-year-old skid trail. The biomass of alder seedlings in the 30-year-old skid trail was not significantly different from its value in the control area, while the biomass of maple seedlings in the 30-year-old skid trail was significantly lower than its value in the control area. The amount of root biomass and total biomass of both alder and maple species in the 30-year-old skid trail was significantly lower than that in the control area. There was no significant difference between the values of stem biomass, root biomass, and total biomass of alder and maple seedlings in the 10- and 20-year-old skid trails and the control area, but in the 30-year-old skid trails, the stem biomass and total biomass of alder seedlings were higher than that of maple seedlings (Table 3).

**Figure 1:**
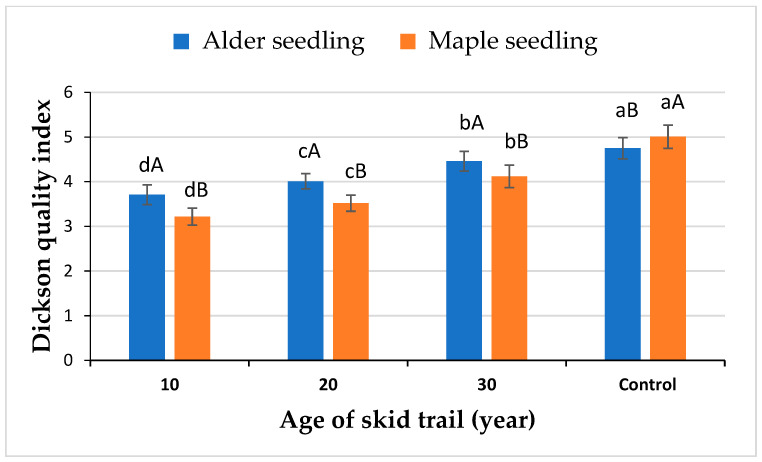
Dickson’s quality index for alder and maple seedlings (Average ± standard deviation) in study treatments. Capital case letters refer to the comparison made between seedling species using independent samples *t*-test at α = 0.05 in each age class. Lowercase letters refer to the comparison made among the three ages of skid trails and control using Duncan’s test at *p* = 0.05 in each species.

Seedling quality index of both alder and maple seedlings species increased with increasing the time elapsed since the skid trail was used (Figure 1). The highest value of the seedling quality index of both alder and maple seedlings species was in the control area. The seedling quality index of both alder and maple seedlings species in the control area was not significantly different from their values in the 30-year-old skid trails. Also, the seedling quality index of both the alder and maple seedlings species in the 20-year-old skid trail was not significantly different from their values in the 10-year-old skid trail. Alder seedling quality index was higher than maple seedling quality index in 10- and 20-year-old skid trails, but there was no significant difference in 30-year-old skid trails and the control area (Figure 1).

The results of the correlation analysis showed that all growth characteristics, biomass, and quality index of both alder and maple seedlings have a significant negative relationship with soil bulk density and with soil penetration resistance, and a significant positive relationship with soil porosity, moisture, and organic matter (Table 4). The amount of soil nitrogen had a significant positive correlation with the length of the secondary roots and the biomass of the belowground organs of both alder and maple seedlings species. The acidity (pH) of the soil had a significant positive correlation with the length of the main root and the root diameter of the seedlings of both alder and maple species. Growth characteristics, biomass, and quality index of seedlings of both alder and maple seedling species had the highest correlation value with soil penetration resistance.

The results of analysis of variance showed that among the investigated architectural indices, the index of root-to-stem ratio and the ratio of penetration to root length of seedlings of both alder and maple species were affected by the studied treatments (Table 5). The indices of ratio of lateral to main root and the ratio of root biomass of both alder and maple seedlings species were not significantly different from each other in different treatments.

**Figure 2:**
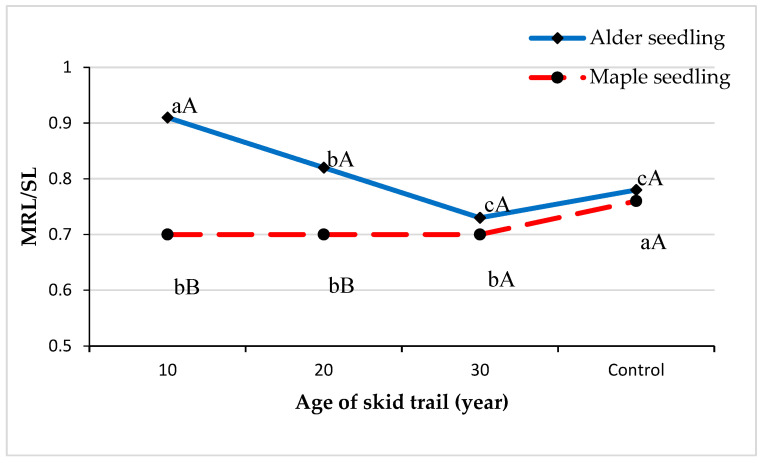
Average ratio of main root length to stem length (MRL/SL) in treatments and seedling species. Capital case letters refer to the comparison made between seedling species using independent samples *t*-test at *p* = 0.05. Lowercase letters refer to the comparison made among the three ages of skid trails and undisturbed area using Duncan’s test at *p* = 0.05.

The average main root-to-stem ratio index (MRL/SL) of alder seedlings on skid trails showed a decreasing trend with increasing age of the skid trail, while this ratio had a constant trend for maple seedlings (Figure 2). In all treatments, the average main root-to-stem ratio index of alder seedlings was higher than that of maple seedlings, although these differences were significant only in 10- and 20-year-old skid trails.

**Figure 3:**
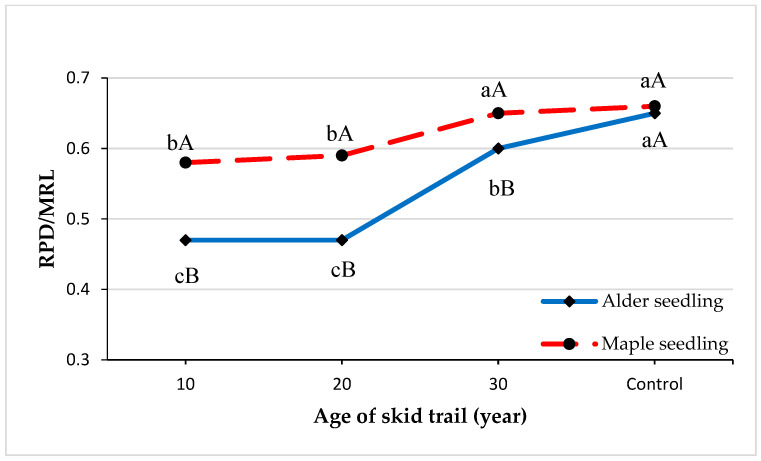
Average ratio of root penetration depth to main root length (RPD/MRL) in treatments and seedling species. Capital case letters (A and B) refer to the comparison made between seedling species using independent samples *t*-test at *p* = 0.05 in each skid trail age. Lowercase letters (a–c) refer to the comparison made among the three ages of skid trail and undisturbed area using Duncan’s test at *p* = 0.05 for each species.

The average index of penetration ratio to root length of seedlings of both alder and maple species on skid trails showed an increasing trend with increasing age of the skid trails (Figure 3). In all treatments, the average index of penetration ratio to the root length of maple seedlings was higher than that of alder seedlings, although these differences were significant only on the skid trails.

## 3. Discussion

The results of this research showed that all the soil characteristics of the 10- and 20-year-old skid trails were significantly different from the control area (without compaction), except for soil moisture in the 20-year-old skid trail. The values of soil bulk density, total porosity, penetration resistance, moisture, and acidity of the soil of 30-year-old skid trails were not significantly different from the control area, while the values of organic matter and soil nitrogen of the 30-year-old skid trails were significantly lower than those in the control area. Changes over time in the physical and chemical characteristics of the impacted soil were noted. The first consideration that can be emphasized is that the natural change in soil properties years after logging is a positive process because it triggers a rehabilitation of the physical and chemical characteristics of the soil. Secondly, this process is a long-term one and it requires a period of more than 30 years to fully restore all soil properties that influence the growth of seedlings. Numerous authors indicate that some form of recovery did occur over a long period [16,40,41,42,43,44,45]. Sohrabi et al. [45] showed that the negative effects of soil compaction can be seen even 20 years after logging by skidder. Furthermore, Kiumarsi et al. [46] showed that soil physicochemical properties need more than 27 years to return to their original state. Research by Alexander [47] showed that even 40 years after timber harvesting, soil compaction was significantly greater in areas which underwent machine traffic compared to areas which did not.

We found that the growth characteristics of alder and maple seedlings were influenced by the place of growth. The minimum growth profile was observed on skid trails and the maximum was observed in the control area. Jourgholami et al. [27] reported that all the variables related to the morphology and architecture of maple seedlings decrease with the increase in soil penetration resistance. As shown in [3], in some cases, only three passes of a logging machine on a skid trail create soil characteristics that are detrimental to seedling growth and long-term habitat productivity. The length and diameter of the main root and the root penetration depth of both alder and maple seedling species on the 30-year-old skid trails were lower than their similar values in the control area, while the averages of other growth characteristics on the 30-year-old skid trails were not significantly different from the control area. In the conditions of soil compaction, the root system of the seedlings of these two species is more sensitive than the growth of the aboveground organs, and they need a longer time to recover. Soil compaction had a negative effect on the growth of both alder and maple seedlings. The amount of growth of aboveground organs and the root system of seedlings of both species were negatively affected by the conditions of the skid trails. Based on these observations, higher soil bulk density was associated with thinner roots, shorter main and lateral roots, and lower penetration depth. According to the results of this research on alder and maple seedlings, adverse effects have also been reported in other seedling species [1,32,33,34,48,49].

In the 10-year-old skid trails, only the root growth of alder seedlings was higher than that of maple seedlings, while in the 20- and 30-year-old skid trails, in addition to root growth, the growth of all vegetative characteristics of alder seedlings was higher than that of maple seedlings. These findings show that alder species could be more suitable than maple species in the selection of seedlings for an active recovery of skid trails. The effects of soil compaction on plants are related to soil type [50] and forest growth system [3]. In fact, soil compaction induced variations in the morphology of the root system, such as the length and diameter of the main root, and lateral root length, which then also influences the part above the soil [50]. Root length reduction with increasing soil compaction was observed for many plant species including trees [51]. In this mixed broadleaf forest, even after 30 years of soil compaction, the biomass of seedlings grown on skid trails was less than the control area. Jourgholami et al. [34] investigated the effects of soil compaction on the morphology, growth, and architecture of tall oak seedlings (*Quercus castaneifolia* C.A. Mey.) in laboratory conditions. Their results showed that both aboveground and belowground seedling traits, including size and biomass, were affected by soil compaction. At the highest intensity of soil compaction, the size and growth of seedlings decreased by 50% compared to the control group. Negative effects were usually more severe below ground (i.e., root system length and biomass) than aboveground. According to the findings of Bassett et al. [51], excessive soil compaction, reducing porosity, and the connection between pores limits the access of plant roots to oxygen. As a result, this hinders the process of gas, water, and heat exchange, and ultimately can reduce seedling root growth and even prevent the growth of lateral roots.

The quality of the seedlings grown on skid trails were lower than the quality of seedlings grown in the control area, although there was no significant difference between the quality of the seedlings grown on the 30-year-old skid trails and those grown in the control area. The quality of alder seedlings in all treatments was higher than the quality of maple seedlings. The results showed that all growth characteristics, biomass, and quality index of seedlings have a significant negative relationship with soil bulk density and soil penetration resistance, and a significant positive relationship with soil porosity, moisture, and organic matter. Growth characteristics, biomass, and quality index of seedlings of both alder and maple species had the highest negative correlation value with soil penetration resistance. Increasing soil penetration resistance causes a decrease in root growth system and also decreases the rooting depth of plants, especially young plants such as seedlings, which leads to a decrease in the absorption of water and nutrients by the plant, and as a result decreases the growth and amount of biomass of seedlings. Dickson’s index has a direct relationship with root biomass, so as root biomass decreases due to increased soil penetration resistance, Dickson’s index also decreases.

The complex phenomenon of compaction produces a decrease in certain positive characteristics for seedling establishment [52]. The consequences are not only on this aspect but can also produce a modification of the microbiome and microfauna [1,36,53,54,55]. Soil compaction had a significant effect on the architectural indices of seedlings, including the ratio of root to stem length and the ratio of root penetration to root length. The index value of root length to stem of alder seedlings was higher than that of maple seedlings, but the index value of root penetration ratio to root length of maple seedlings was higher than that of alder seedlings in all treatments. Increasing soil strength may change the proportional allocation of growth between above and underground parts of seedlings [50,51] and reduce the ratio of roots [27]. Plants typically show a wide range of responses to increased soil strength, with plant physiological adaptations influencing their growth, architecture, and water and nutrient use [51]. Removal of wood or biomass due to timber harvesting in forests affects the forest soil and natural recovery, which is especially important for maintaining biodiversity. Some good practices recommend adding utilization residues, such as branches and tops, on the skid trails to favor the recovery of compacted areas from the passage of the logging vehicles as well as the addition of litter or mulch at the end of harvesting operations to improve the recovery of compacted soil [52,53]. These strategies to diminish the effects of soil compaction are likely to be related to the creation of an environment that favors not only soil dynamics and functional processes, but also the maintenance of soil biological activity [14,54,55], which can also improve the soil physical characteristics. Monitoring the effects of forest operations is a crucial requirement for sustainable forest management [33].

## 4. Materials and Methods

### 4.1. Study Area

This study was conducted in parcel No. 47 from series No. 1 in the forests of Asalem in the north of Iran (Hyrcanian forests). This series is located in watershed No. 7 in the Guillan province. The geographic coordinates of this series are 48° 48′ to 48° 52′ east longitude, and 37° 38′ to 37° 42′ north latitude. The area of parcel 47 is 41 hectares, the height above sea level is from 1150 to 1350 m, and the general direction of the land slope is northwest. The climate of the region is in the humid group based on the De Martonne’s humidity coefficient. Based on the 10 year statistics (2012–2022), the average annual rainfall is 924 mm and the average annual temperature is around 10.2 °C. The soil is silica parent rock, which is a forest brown soil type with sandy loam texture, has proper drainage, acidic pH between 5.5 and 2.6, and is covered with a layer of litter 2 to 7 cm deep. The studied forest is a mixed beech type with an uneven aged structure. The abundance of tree species in the studied parcel is, respectively, Beech (*Fagus orientalis* Lipsky) 43.4%, hornbeam (*Carpinus betulus* L.) 23.5%, velvet maple (*Acer velutinum* Boiss) 10.6%, Cappadocian maple (*Acer cappadocicum* Gled.) 9.5%, alder (*Alnus subcordata* C.A.M.) 8.9%, and the abundance of other tree species is 1.4%, which mainly includes oak (*Quercus castaneifolia* C.A.M.), ash (*Fraxinus coriarifolia* Scheel), lime tree (*Tilia begonifolia* Stev.), elm (*Ulmus glabra* Huds.), and Caucasian elm (*Zelkova carprinifolia* Diopp.).

The forest canopy cover is 80–100%, the density of trees is 290 stems/ha, the basal area of trees is 19.1 m^2^/ha, and the standing volume of the trees is 238 m^3^/ha. In these forests, the forest management method is close to nature (single tree selection) with a logging period of 10 years. The logging system is generally cut to length, and the method of log extraction is usually ground skidding using rubber wheel skidders equipped with winch cables. Marked trees were cut and processed with a chainsaw, and then extraction was performed using Timberjack C450 rubber wheel skidder. The weight of the logging machine is 9.8 tons, the engine power is 177 hp (132 kW), and its length and width are 6.4 and 3.8 m, respectively. The length of logs was 5.2 and 7.8 m, and the maximum length of the winching cable is 50 m. The width of skid trails was 4 m, their average distance from each other was 140 m, and their density was 24.5 m per hectare.

### 4.2. Research Design and Data Collection

In order to evaluate the soil recovery process, growth, and qualitative condition of alder and maple seedlings on soils compacted due to the skidding operation in the studied forest, skid trails last used 10, 20, and 30 years ago were identified (SKT10, SKT20, and SKT30), with 2 replications of each (6 skid trails). These three periods were indicated as the age of the skid trail. This study was retrospective. The age of skid trails and the type of logging machines used were identified through the study forest management plan booklet. The beginning of the selected logging routes was embanked and blocked after the completion of the logging operation, and there were no traffic of logging machines. The skidding direction was downhill in all of the selected skid trails. The length of the skid trails was about 150 m. The maximum slope of skid trails was 25%. To minimize the impact of the machine traffic and slope variation, the soil and seedling samples were taken from the middle segments of skid trails with a consistent 10% incline. Sampling on the skid trails was performed using sample plots (SKT-P) of 40 m^2^ (4 m × 10 m) with a random starting point and a regular distance of 2 m intervals with three replications in each skid trail [55] (3 age × 2 replication × 3 SKT-P = 18 SKT-P). The position of the plots on the skid trail was such that the width of the plots (4 m) covered the width of the skid trail and the length of the plots (10 m) extended along the skid trail. For each skid trail sample plot (SKT-P), 1 plot (2 m × 2 m) at a distance of 50 m from the skid trail in the untouched parts of the forest were considered as control plots (C-P) (3 control plots for each skid trail).

#### 4.2.1. Soil Sampling

In each skid-trail sample plot, 4 transects were established at a distance of 2 m from each other and perpendicular to the skid trails. The number of 3 transects in each skid-trail sample plot was randomly selected. Two soil samples were collected from each selected transect (one from left skidder wheel rut and one from right skidder wheel rut) using metal cylinders with an inner diameter of 5 cm and a height of 10 cm from the soil surface, after removal of the litter. They were placed inside a plastic bag and transferred to the soil laboratory for measurement of the bulk density, porosity, moisture, organic matter, nitrogen, and soil acidity. Also, from each control plot (C-P), one soil sample was taken from the center of the plot and transferred to the soil laboratory. Soil penetration resistance (SPR) was measured using a pocket penetrometer model Eijkelkamp, Zevenaar, Netherlands, at 0, 5, and 10 cm soil depths, on skidder wheel rut, 2 samples in each transect (1 from left skidder wheel rut and 1 from right skidder wheel rut), and 4 samples of SPR were measured at random points inside the control plot at equal soil depths. The rod of the penetrometer was placed perpendicular to the soil surface and was pressed into the flat surface with a gentle and smooth constant force until the entire length of the rod (6.35 mm) penetrated into the soil, and then the value of SPR was read from the indicator ring. The SPR value of each soil sample was calculated from the average of the SPR measurements at three depths of that soil sample.

The bulk density of the soil, which is the ratio of the dry weight of the soil to the volume of the soil sample (after drying the soil in the oven for 24 h at 105 °C), was calculated by Equation (1).

BD = WD/VC(1)

In Equation (1), BD is the bulk density in g/cm^3^, WD is the dry weight of the soil in g, and VC is the volume of the cylinder of the soil sample in cm^3^.

The total soil porosity was determined using this Equation (2) [33]:

TP = (1 − BD/2.65) × 100(2)

In Equation (2), TP is the total porosity of the soil in percent, BD is the soil bulk density in g/cm^3^, and 2.65 is the density of soil particles (g/cm^3^) measured using a pycnometer on the same soil samples used to determine the bulk density.

The following characteristics were also determined: soil moisture content using the weight method; soil acidity (pH) in a 1:1 ratio of water to soil [56]; soil organic matter using the Walkley and Black method [57]; soil nitrogen using the Kjeldahl method [58].

#### 4.2.2. Seedling Sampling

Some healthy seven-year-old seedling of alder and maple species (closest to the soil samples), which had grown naturally years after logging operations, were identified in each skid-trail sample plot (3 alder seedlings and 3 maple seedlings in each skid trail). The age of the seedlings was determined using the phyllotaxy method [59]. The following variables were measured for each seedling: physical characteristics including seedling height (SH), stem length (SL), stem diameter (SD), main root length (MRL), main root diameter (MRD), lateral root length (LRL), and root penetration depth (RPD); growth characteristics including total dry biomass (TDB), stem dry biomass (SDB), and root dry biomass (RDB); and architectural characteristics including root to stem ratio (MRL/SL), lateral to main root ratio (LRL/MRL), root penetration depth to root length ratio (RPD/MRL), and root to stem dry biomass ratio (RDB/SDB). Stem and root diameters were measured with a vernier caliper (Insize Model 1205, Derio, Spain) 5 cm above and below the soil surface, respectively. The vertical distance from the root tip to the soil surface was measured using a metal ruler as the root penetration depth. The dry weight of seedlings was obtained after drying at 70 °C until constant weight was reached [16]. Dickson quality index (DQI) was calculated from Equation (3) [60].

DQI = (TDB (g))/(SH(cm)/SD(mm) + (SDB (g))/(RDB (g)))(3)

The Dickson Quality Index (DQI) assesses seedling vigor and survival potential. Higher DQI values indicate improved survival prospects. Given its consideration of biomass distribution and key seedling parameters, DQI is a suitable indicator of seedling quality [60].

### 4.3. Statistical Analysis

Statistical analysis on collected data were carried out using SPSS version 20 software (Chicago, IL, USA). The normality of data distribution was checked using the Kolmogorov–Smirnov test (*p* = 0.05). The homogeneity of variance between treatments was tested and confirmed using Levene’s test (*p* = 0.05). The effect of the treatments (skid trails with different ages and the control area) on soil properties and seedling characteristics was analyzed using ANOVA. Duncan’s test was used to find the difference between treatment means at *p* ≤ 0.05. An independent samples *t*-test was used to compare the average growth characteristics and biomass of alder and maple seedlings. The relationship between soil properties and seedling characteristics was determined using the Pearson correlation test.

## 5. Conclusions

The study investigated soil conditions (physical and chemical properties) on skid trails after 10, 20, and 30 years, and assessed the impact of soil compaction on alder and maple seedling growth, yield, morphology, and quality. Results indicated that compacted soil typically recovers within slightly more than two decades. However, restoring alder and maple seedling growth and quality to pre-logging conditions requires a significantly longer period. Therefore, the accurate design of skid trails before the implementation of logging operations, the addition of logging residuals on skid trails before skidding operations, and the rapid recovery of compacted soils through best management practices (BMP) methods are necessary in these forests.

Alder seedlings performed better than maple seedlings on skid slopes, and seedlings of both alder and maple species are suitable for bioengineering operations to recover the properties of soils compacted by forest machine traffic. Although alder seedlings had a better performance than maple seedlings on skid trails, we can conclude and recommend, based on our findings, that seedlings of both alder and maple are suitable for implementing bioengineering operations to recover the properties of compacted soils caused by the traffic of logging machines.

## Figures and Tables

**Table 1:** Physical and chemical properties of skid trail soil (Average ± standard deviation).

Soil Properties	Age of Skid Trail (Year)				*F* Value
	10	20	30	Control	
Bulk density (g/cm^3^)	1.26 ± 0.05 a	1.20 ± 0.04 a	1.10 ± 0.04 b	1.02 ± 0.05 b	136.4 **
Porosity (%)	52.4 ± 1.65 b	54.7 ± 1.82 b	58.5 ± 1.94 a	61.5 ± 3.01 a	107.3 **
Soil penetration resistance (MPa)	0.46 ± 0.05 a	0.38 ± 0.06 b	0.29 ± 0.04 c	0.26 ± 0.05 c	215.1 **
Moisture (%)	37.8 ± 1.70 b	40.1 ± 1.88 a b	44.0 ± 2.02 a	44.5 ± 2.10 a	33.9 **
Organic matter (%)	2.64 ± 1.08 d	2.96 ± 0.07 c	3.70 ± 0.07 b	4.08 ± 0.11 a	59.9 **
Nitrogen (%)	0.22 ± 0.04 c	0.26 ± 0.03 c	0.39 ± 0.11 b	0.51 ± 0.05 a	45.7 **
C/N ratio	12.00 ± 0.07 a	11.38 ± 0.06 a	9.49 ± 0.06 b	8.00 ± 0.08 c	68.4 **
pH (1:1 H_2_O)	5.50 ± 0.06 b	5.56 ± 0.06 b	5.79 ± 0.11 a	5.88 ± 0.10 a	65.7 **

**: Significant at *p* = 0.01. Different letters after mean indicate significant difference using Duncan’s test at α = 0.05 in each row.

**Table 2:** Growth characteristics of Alder and Maple seedlings on skid trails and control (Average ± standard deviation).

Seedling Growth Characteristics	Seedling Species	Age of Skid Trail (Year)				*F* Value
		10	20	30	Control	
Stem length (cm)	Alder	49.0 ± 2.5 cA	57.0 ± 3.3 bA	68.5 ± 3.3 aA	69.0 ± 3.1 aA	77.0 **
	Maple	48.8 ± 3.0 bA	50.2 ± 3.3 bB	57.6 ± 3.5 aB	60.2 ± 2.7 aB	79.4 *
Stem height (cm)	Alder	46.1 ± 2.4 cA	54.5 ± 2.6 bA	65.6 ± 2.7 aA	66.7 ± 3.2 aA	67.2 **
	Maple	45.0 ± 2.1 cA	47.3 ± 2.3 bB	54.5 ± 3.0 aB	57.2 ± 2.8 aB	96.1 **
Stem diameter (mm)	Alder	3.75 ± 0.6 dA	4.10 ± 0.5 cA	4.69 ± 0.7 aA	4.71 ± 0.5 aA	29.7 **
	Maple	3.71 ± 0.6 dA	3.91 ± 0.6 cB	4.31 ± 0.8 bB	4.45 ± 0.5 aB	61.5 **
Main root length (cm)	Alder	44.7 ± 2.2 cA	46.9 ± 2.8 cA	50.0 ± 2.2 bA	54.3 ± 2.5 aA	39.5 **
	Maple	34.7 ± 2.6 cB	35.1 ± 2.5 cB	40.2 ± 2.5 bB	45.6 ± 2.3 aB	23.7 **
Main root diameter (mm)	Alder	3.80 ± 0.5 dA	4.20 ± 0.6 cA	4.72 ± 0.6 bA	4.88 ± 0.7 aA	99.4 **
	Maple	3.81 ± 0.6 dA	4.07 ± 0.5 cB	4.50 ± 0.5 bB	4.62 ± 0.7 aB	112.4 **
Lateral root length (cm)	Alder	49.3 ± 2.6 bA	50.5 ± 3.2 bA	57.1 ± 3.3 aA	59.0 ± 3.2 aA	26.9 **
	Maple	36.4 ± 2.5 bB	37.9 ± 2.2 bB	50.9 ± 2.7 aB	51.5 ± 3.1 aB	23.7 **
Root penetration length (cm)	Alder	20.9 ± 2.7 cA	21.7 ± 2.2 cA	30.1 ± 2.5 bA	35.2 ± 2.0 aA	44.0 **
	Maple	20.2 ± 2.6 cA	20.6 ± 2.5 cA	26.1 ± 2.4 bB	30.0 ± 2.0 aB	31.5 **

*: Significant at *p* < 0.05. **: Significant at *p* < 0.01. Capital case letters (A and B) refer to the comparison made between seedling species using independent samples *t*-test at *p* = 0.05 in each age class. Lowercase letters (a–d) refer to the comparison made among the three ages of skid trails and control using Duncan’s test at *p* = 0.05 in each species.

**Table 3:** Biomass of Alder and Maple seedlings on skid trails and control (Average ± standard deviation).

Biomass of Seedlings	Seedling Species	Age of Skid Trail (Year)				*F* Value
		10	20	30	Control	
Stem biomass (g)	Alder	33.0 ± 2.0 bA	35.2 ± 2.2 bA	41.5 ± 2.4 aA	43.2 ± 2.3 aA	21.4 **
	Maple	35.5 ± 2.1 bA	34.0 ± 2.6 bA	35.2 ± 2.1 bB	42.4 ± 2.5 aA	17.5 **
Root biomass (g)	Alder	22.2 ± 1.7 cA	24.0 ± 2.1 b cA	26.8 ± 2.0 bA	30.7 ± 2.0 aA	28.2 **
	Maple	21.0 ± 1.5 bA	22.8 ± 1.6 bA	23.2 ± 1.8 bA	29.2 ± 2.0 aA	14.8 **
Total biomass (g)	Alder	55.5 ± 2.4 dA	59.2 ± 2.6 cA	68.3 ± 3.1 bA	73.9 ± 3.1 aA	54.7 **
	Maple	53.5 ± 2.3 cA	56.8 ± 2.5 bA	58.4 ± 2.6 bB	71.6 ± 2.8 aA	51.0 **

**: Significant at *p* = 0.01. Capital case letters (A and B) refer to the comparison made between seedling species using independent samples *t*-test at *p* = 0.05 in each age class. Lowercase letters (a–d) refer to the comparisons made among the three ages of skid trails and the control using Duncan’s test at *p* = 0.05 in each species.

**Table 4:** Pearson correlation coefficient (R-value) between soil properties and characteristics of alder and maple seedlings (BD: bulk density, TP: total porosity, SPR: soil penetration resistance, SH: seedling height, SL: stem length, SD: stem diameter, MRL: main root length, MRD: main root diameter, LRL: lateral root length, RPD: root penetration depth, SDB: stem dry biomass, RDB: root dry biomass, TDB: total dry biomass, and DQI: Dickson quality index).

Soil Physical and Chemical Properties	Seedling Species	SH (cm)	SL (cm)	SD (mm)	MRL (cm)	MRD (mm)	LRL (cm)	RPD (cm)	SDB (g)	RDB (g)	TDB (g)	DQI
BD (g/cm^3^)	Alder	−0.49 **	−0.48 **	−0.45 **	−0.83 **	−0.58 **	−0.56 **	−0.72 **	−0.49 **	−0.51 **	−0.59 **	−0.66 **
	Maple	−0.55 **	−0.58 **	−0.48 **	−0.85 **	−0.58 **	−0.55 **	−0.79 **	−0.53 **	−0.55 **	−0.64 **	−0.70 **
TP (%)	Alder	0.55 **	0.40 *	0.39 *	0.52 **	0.33 *	0.46 **	0.69 **	0.59 **	0.66 **	0.70 **	0.63 **
	Maple	0.57 **	0.41 *	0.37 *	0.54 **	0.35 *	0.53 **	0.75 **	0.60 **	0.72 **	0.76 **	0.65 **
SPR (KPa)	Alder	−0.61 **	−0.53 **	−0.41 *	−0.84 **	−0.57 **	−0.88 **	−0.89 **	−0.76 **	−0.81 **	−0.85 **	−0.74 **
	Maple	−0.63 **	−0.59 **	−0.44 *	−0.88 **	−0.59 **	−0.86 **	−0.90 **	−0.77 **	−0.80 **	−0.86 **	−0.72 **
MC (%)	Alder	0.40 *	0.40 *	0.39 *	0.51 **	0.37 *	0.37 *	0.43 *	0.56 **	0.59 **	0.60 **	0.48 **
	Maple	0.41 *	0.43 *	0.38 *	0.49 **	0.34 *	0.41 *	0.45 *	0.57 **	0.58 **	0.61 **	0.51 **
OC (%)	Alder	0.45 *	0.40 *	0.38 *	0.44 *	0.37 *	0.53 **	0.59 **	0.53 **	0.57 **	0.62 **	0.56 **
	Maple	0.47 *	0.39 *	0.39 *	0.46 *	0.36 *	0.55 **	0.60 **	0.55 **	0.60 **	0.64 **	0.59 **
N (%)	Alder	0.14	0.10	0.22	0.12	0.19	0.37 *	0.11	0.18	0.36 *	0.22	0.19
	Maple	0.12	0.13	0.20	0.11	0.17	0.35 *	0.14	0.15	0.35 *	0.19	0.21
pH	Alder	0.08	0.10	0.11	0.37 *	0.10	0.33 *	0.10	0.14	0.37 *	0.18	0.20
	Maple	0.09	0.08	0.10	0.35 *	0.13	0.39 *	0.09	0.15	0.35 *	0.14	0.15

* Significant at *p* = 0.05 and ** Significant at *p* = 0.01.

**Table 5:** Results of ANOVA for effects of treatments on alder and maple seedlings’ architecture.

Seedling Architecture Indices	Ratio of MRL to SL	Ratio of LRL to MRL	Ratio of RPD to MRL	Ratio of Root Biomass to Total Biomass
Alder seedling	0.000 **	0.342	0.000 **	0.507
Maple seedling	0.045 *	0.359	0.000 **	0.482

MRL = main root length; SL = stem length; LRL = lateral root length; RPD = root penetration depth; *: Significant at *p* = 0.05 and **: Significant at *p* = 0.01

## Data Availability

Data are available from the corresponding author upon reasonable request.

## References

[1] Cambi M., Hoshika Y., Mariotti B., Paoletti E., Picchio R., Venanzi R., Marchi E. (2017). Compaction by a forest machine affects soil quality and *Quercus robur* L. seedling performance in an experimental field. For. Ecol. Manag..

[2] Robinson D., Emmett B., Reynolds B., Rowe E., Spurgeon D., Keith A., Lebron I., Hockley N., Hester R., Harrison R. (2012). Soil Natural Capital and Ecosystem Service Delivery in a World of Global Soil Change. Soils Food Secur..

[3] Picchio R., Mederski P.S., Tavankar F. (2020). How and how much, do harvesting activities affect forest soil, regeneration and stands?. Curr. For. Rep..

[4] Schweier J., Magagnotti N., Labelle E.R., Athanassiadis D. (2019). Sustainability impact assessment of forest operations: A review. Curr. For. Rep..

[5] Ganatsios H.P., Tsioras P.A., Papaioannou A.G., Blinn C.R. (2021). Short term impacts of harvesting operations on soil chemical properties in a mediterranean oak ecosystem. Croat. J. For. Eng..

[6] Page-Dumroese D., Jurgensen M., Elliot W., Rice T., Nesser J., Collins T., Meurisse R. (2000). Soil quality standards and guidelines for forest sustainability in northwestern North America. For. Ecol. Manag..

[7] Hartmann M., Niklaus P.A., Zimmermann S., Schmutz S., Kremer J., Abarenkov K., Lüscher P., Widmer F., Frey B. (2014). Resistance and resilience of the forest soil microbiome to logging-associated compaction. ISME J..

[8] Grigal D.F. (2000). Effects of Extensive Forest Management on Soil Productivity. For. Ecol. Manag..

[9] Marshall V.G. (2000). Impacts of Forest Harvesting on Biological Processes in Northern Forest Soils. For. Ecol. Manag..

[10] Uusitalo J., Ala-Ilomäki J., Lindeman H., Toivio J., Siren M. (2020). Predicting rut depth induced by an 8-wheeled forwarder in fine-grained boreal forest soils. Ann. For. Sci..

[11] Labelle E.R., Lemmer K.J. (2019). Selected environmental impacts of forest harvesting operations with varying degree of mechanization. Croat. J. For. Eng..

[12] Mederski P.S., Borz S.A., Đuka A., Lazdiņš A. (2021). Challenges in forestry and forest engineering–Case studies from four countries in East Europe. Croat. J. For. Eng. J. Theory Appl. For. Eng..

[13] Agherkakli B., Najafi A., Sadeghi S.H. (2010). Ground based operation effects on soil disturbance by steel tracked skidder in a steep slope of forest. J. For. Sci..

[14] Ampoorter E., Van Nevel L., De Vos B., Hermy M., Verheyen K. (2010). Assessing the effects of initial soil characteristics, machine mass and traffic intensity on forest soil compaction. Forest Ecol. Manag..

[15] Eliasson L. (2005). Effects of forwarder tyre pressure on rut formation and soil compaction. Silva Fenn. Monogr..

[16] Mohieddinne H., Brasseur B., Spicher F., Gallet-Moron E., Buridant J., Kobaissi A., Horen H. (2019). Physical recovery of forest soil after compaction by heavy machines, revealed by penetration resistance over multiple decades. For. Ecol. Manag..

[17] Håkansson R.C. (1994). Reeder Subsoil compaction by vehicles with high axle load—Extent, persistence and crop response. Soil Tillage Res..

[18] Proto A.R., Macrì G., Sorgonà A., Zimbalatti G. (2016). Impact of skidding operations on soil physical properties in southern Italy. Contemp. Eng. Sci..

[19] Eroğlu H., Sariyildiz T., Küçük M., Sancal E. (2016). The effects of different logging techniques on the physical and chemical characteristics of forest soil. Balt. For..

[20] Nazari M., Eteghadipour M., Zarebanadkouki M., Ghorbani M., Dippold M.A., Bilyera N., Zamanian K. (2021). Impacts of logging-associated compaction on forest soils: A meta-analysis. Front. For. Glob. Change.

[21] Basset C., Abou Najm M., Ghezzehei T., Hao X., Daccache A. (2023). How does soil structure affect water infiltration? A meta-data systematic review. Soil. Tillage Res..

[22] Malvar M.C., Silva F.C., Prats S.A., Vieira D.C.S., Coelho C.O.A., Keizer J.J. (2017). Short-term effects of post-fire salvage logging on runoff and soil erosion. For. Ecol. Manag..

[23] Tavankar F., Picchio R., Nikooy M., Jourgholami M., Latterini F., Venanzi R. (2021). Effect of soil moisture on soil compaction during skidding operations in poplar plantation. Int. J. For. Eng..

[24] Bens O., Wahl N.A., Fischer H., Hüttl R.F. (2007). Water infiltration and hydraulic conductivity in sandy cambisols: Impacts of forest transformation on soil hydrological properties. Eur. J. For. Res..

[25] Wagenbrenner J.W., Robichaud P.R., Brown R.E. (2016). Rill erosion in burned and salvage logged western montane forests: Effects of logging equipment type, traffic level, and slash treatment. J. Hydrol..

[26] Hansson L., Šimůnek J., Ring E., Bishop K., Gärdenäs A.I. (2019). Soil compaction effects on root-zone hydrology and vegetation in boreal forest clearcuts. Soil Sci. Soc. Am. J..

[27] Jourgholami M. (2018). Effects of soil compaction on growth variables in Cappadocian maple (*Acer cappadocicum*) seedlings. J. For. Res..

[28] Tavankar F., Nikooy M., Ezzati S., Jourgholami M., Latterini F., Venanzi R., Picchio R. (2022). Long-term assessment of soil physicochemical properties and seedlings establishment after skidding operations in mountainous mixed hardwoods. Eur. J. For. Res..

[29] Jourgholami M., Soltanpour S., Etehadi Abari M., Zenner E.K. (2014). Influence of slope on physical soil disturbance due to farm tractor forwarding in a Hyrcanian forest of northern Iran. iForest..

[30] Warlo H., Zimmermann S., Lang F., Schack-Kirchner H. (2022). Characteristics of Soil Structure and Greenhouse Gas Fluxes on Ten-Year Old Skid Trails with and without Black Alders (*Alnus glutinosa* (L.) *Gaertn*.). Soil Syst..

[31] Schäffer J. (2022). Recovery of Soil Structure and Fine Root Distribution in Compacted Forest Soils. Soil Syst..

[32] Bejarano M.D., Villar R., Murillo A.M., Quero J.L. (2010). Effects of soil compaction and light on growth of *Quercus pyrenaica* Willd. (Fagaceae) seedlings. Soil. Till. Res..

[33] Picchio R., Tavankar F., Nikooy M., Pignatti G., Venanzi R., Lo Monaco A. (2019). Morphology, Growth and Architecture Response of Beech (*Fagus orientalis* Lipsky) and Maple Tree (*Acer velutinum Boiss*.) Seedlings to Soil Compaction Stress Caused by Mechanized Logging Operations. Forests.

[34] Jourgholami M., Khoramizadeh A., Zenner E.K. (2016). Effects of soil compaction on seedling morphology, growth, and architecture of chestnut-leaved oak (*Quercus castaneifolia*). iForest.

[35] Singh S., Malik Z.A., Sharma C.M. (2016). Tree species richness, diversity, and regeneration status in different oak (*Quercus* spp.) dominated forests of Garhwal Himalaya, India. J. Asia-Pac. Biodivers..

[36] Jamshidi R., Jaeger D., Dragovich D. (2018). Establishment of pioneer seedling species on compacted skid tracks in a temperate Hyrcanian Forest, northern Iran. Environ. Earth Sci..

[37] Sabeti H. (1994). Forests, Trees and Shrubs of Iran.

[38] Perez J., Salazar R.C., Stokes A. (2017). An open access database of plant species useful for controlling soil erosion and substrate mass movement. Ecol. Eng..

[39] Liu H., Mao Z., Wang Y., Kim J.H., Bourrier F., Mohamed A., Stokes A. (2021). Slow recovery from soil disturbance increases susceptibility of high elevation forests to landslides. For. Ecol. Manag..

[40] Ezzati S., Najafi A., Rab A., Zenner E.K. (2012). Recovery of Soil Bulk Density, Porosity and Rutting from Ground Skidding Over a 20-Year Period after Timber Harvesting in Iran. Silva Fenn..

[41] Klaes B., Struck J., Schneider R., Schüler G. (2016). Middle-term effects after timber harvesting with heavy machinery on a fine-textured forest soil. Eur. J. Forest Res..

[42] Pousse N., Bonnaud P., Legout A., Darboux F., Ranger J. (2022). Forest Soil Penetration Resistance Following Heavy Traffic: A 10-YearField Study. Soil Use Manag..

[43] Kabzems R., Haeussler S. (2005). Soil properties, aspen, and white spruce responses 5 years after organic matter removal and compaction treatments. Can. J. For. Res..

[44] Maloney K.O., Garten C.T., Ashwood T.L. (2008). Changes in soil properties following 55 years of secondary Forest succession at fort Benning, Georgia, U.S.A. Restor. Ecol..

[45] Sohrabi H., Jourgholami M., Tavankar F., Venanzi R., Picchio R. (2019). Post-Harvest Evaluation of Soil Physical Properties and Natural Regeneration Growth in Steep-Slope Terrains. Forests.

[46] Kiumarsi F., Jourgholami M., Jafari M., Lo Monaco A., Venanzi R., Picchio R. (2024). Restoring soil properties in the Hyrcanian forests from machine induced compaction: Reforestation of N2-fixing black alder (*Alnus glutinosa* (L.) Gaertn.). Land Degrad. Dev..

[47] Alexander A.B. (2012). Soil compaction on skid trails after selective logging in moist evergreen forest of Ghana. Agr. Biol. J. N. Am..

[48] Gomez A., Powers R.F., Singer M.J., Horwath W.R. (2002). Soil compaction effects on growth of young ponderosa pine following litter removal in California’s Sierra Nevada. Soil Sci. Soc. Am. J..

[49] Brais S. (2001). Persistence of soil compaction and effects on seedling growth in Northwestern Quebec. Soil Sci. Soc. Am. J..

[50] Alameda D., Villar R. (2012). Linking root traits to plant physiology and growth in Fraxinus angustifolia Vahl. seedlings under soil compaction conditions. Environ. ExBot.

[51] Bassett I.E., Simcock R.C., Mitchell N.D. (2005). Consequences of soil compaction for seedling establishment: Implications for natural regeneration and restoration. Austral Ecol..

[52] Flores Fernández L., Rubin P., Hartmann H., Puhlmann K. (2019). von Wilpert, Initial recovery of soil structure of a compacted forest soil can be enhanced by technical treatments and planting. For. Ecol. Manag..

[53] Jourgholami M., Khoramizadeh A., Lo Monaco A., Venanzi R., Latterini F., Tavankar F., Picchio R. (2021). Evaluation of Leaf Litter Mulching and Incorporation on Skid Trails for the Recovery of Soil Physico-Chemical and Biological Properties of Mixed Broadleaved Forests. Land.

[54] Battigelli J.P., Spence J.R., Langor D.W., Berch S.M. (2004). Short-term impact of forest soil compaction and organic matter removal on soil mesofauna density and oribatid mite diversity. Can. J. For. Res..

[55] Sohrabi H., Jourgholami M., Lo Monaco A., Picchio R. (2022). Effects of Forest Harvesting Operations on the Recovery of Earthworms and Nematodes in the Hyrcanain Old-Growth Forest: Assessment, Mitigation, and Best Management Practice. Land.

[56] Thomas G.W., Sparks D.L. (1996). Soil pH and soil acidity. Methods of Soil Analysis, Part 3—Chemical Methods.

[57] Nelson D.W., Sommers L.E., Sparks D.L. (1996). Total Carbone, organic Carbone, and organic matter. Methods of Soil Analysis, Part 3—Chemical Methods.

[58] Bremner J.M., Sparks D.L. (1996). Nitrogen-total. Methods of Soil Analysis, Part 3—Chemical Methods.

[59] Kuhlemeier C. (2007). Phyllotaxis. Trends Plant Sci..

[60] Nyoka B.I., Kamanga R., Njoloma J., Jamnadass R., Mng’omba S., Muwanje S. (2018). Quality of Tree Seedlings Produced in Nurseries in Malawi: An Assessment of Morphological Attributes. For. Trees Livelihoods.

